# Supplementary material for: YB1 Is a Major Contributor to Health Disparities in Triple Negative Breast Cancer
Source: Cancers (Basel). 2021 Dec 14;13(24):6262. doi: 10.3390/cancers13246262 (PMC8699660; doi:10.3390/cancers13246262)
Supplement: Supplementary file 1 [file cancers-13-06262-s001.zip › cancers-1482740-supplementary.pdf]

Supplementary Materials: YB1 Is a Major Contributor to Health Disparities in Triple Negative Breast Cancer

Priyanka Shailendra Rana, Wei Wang, Akram Alkrekshi, Vesna Markovic, Amer Khiyami, Ricky Chan, Adam Perzynski, Natalie Joseph and Khalid Sossey-Alaoui

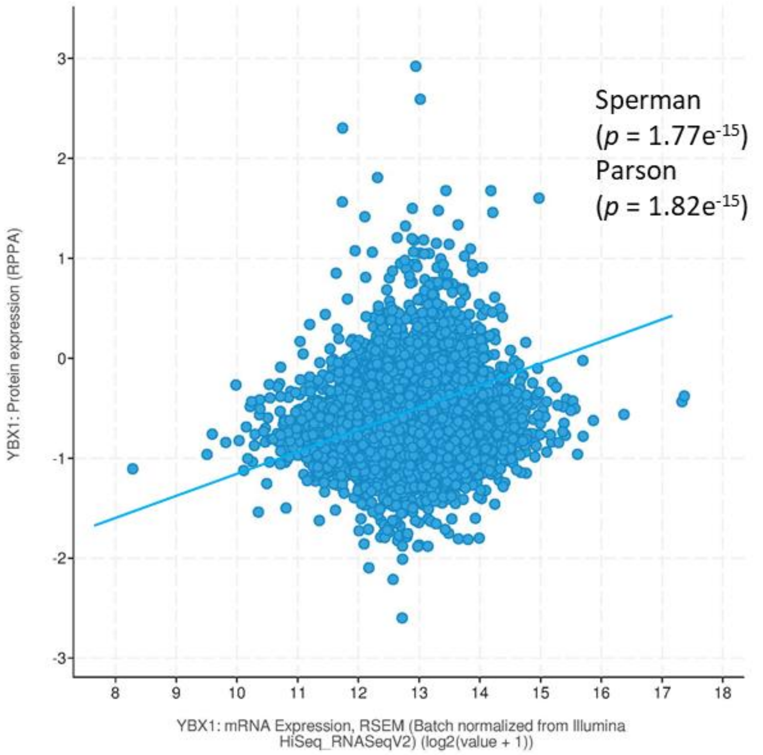

Figure S1. Correlation between YB1 mRNA and protein levels in the PanCancer TCGA data set. mRNA levels are plotted as log2 RSEM values batch normalized from Illumina HiSeq RNASeq data, while protein levels are plotted as RPPA.

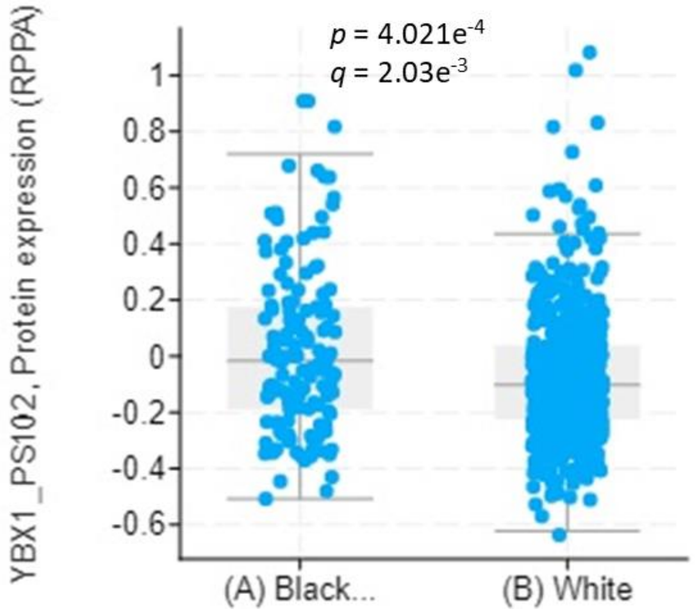

Figure S2. Comparison of YB1 protein phosphorylation levels, based on RPPA, between AA and CA BC patients from the TCGA BRCA PanCancer Atlas cohort. YB1 protein phosphorylation levels are significantly higher (Wilcoxon) in AA compared to CA TNBC tumors.

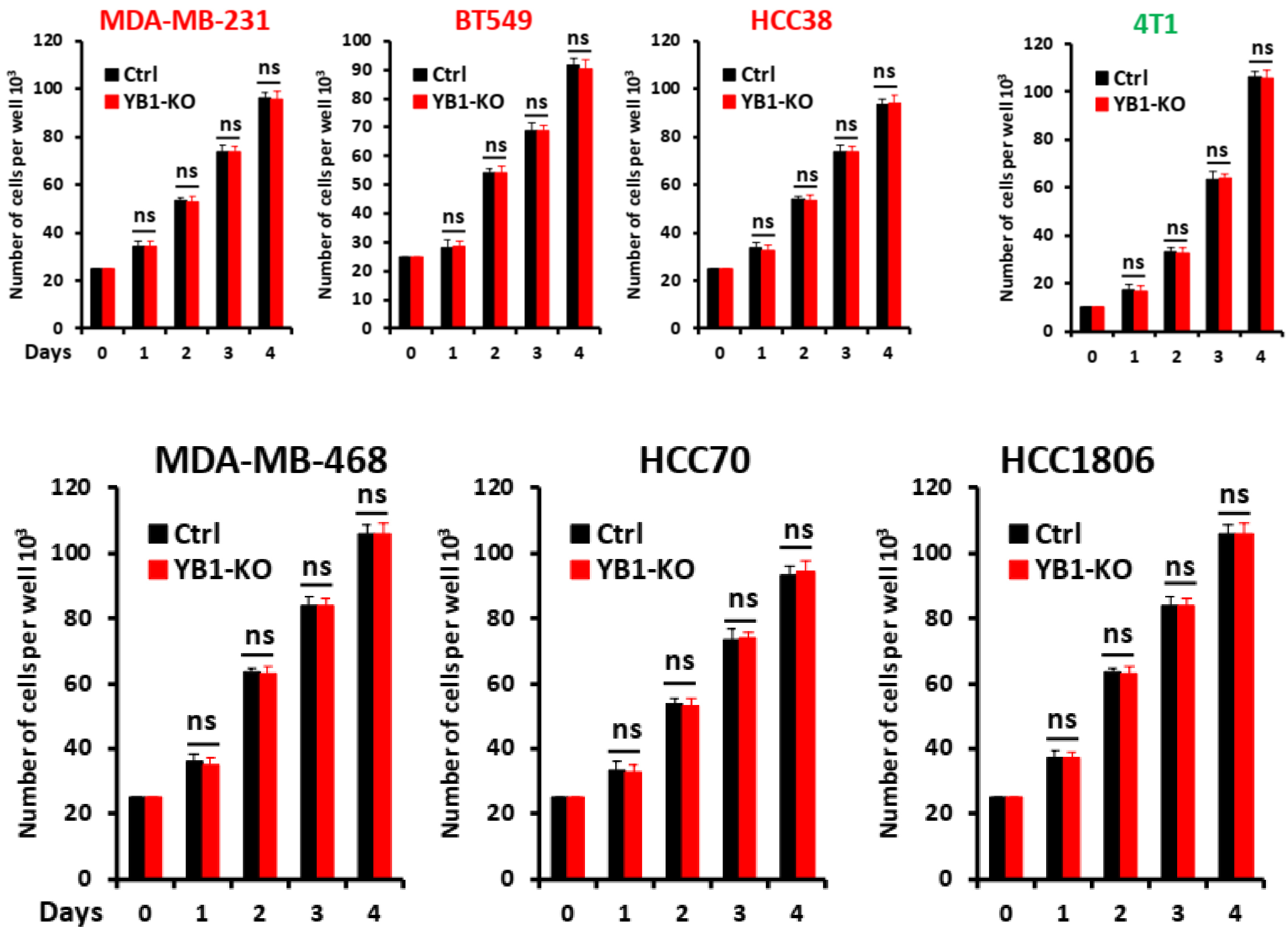

Figure S3. Cell proliferation over 5 days of the indicated TNBC cell lines and their YB1-KO derivatives. Viable cell were Identified with PI staining.

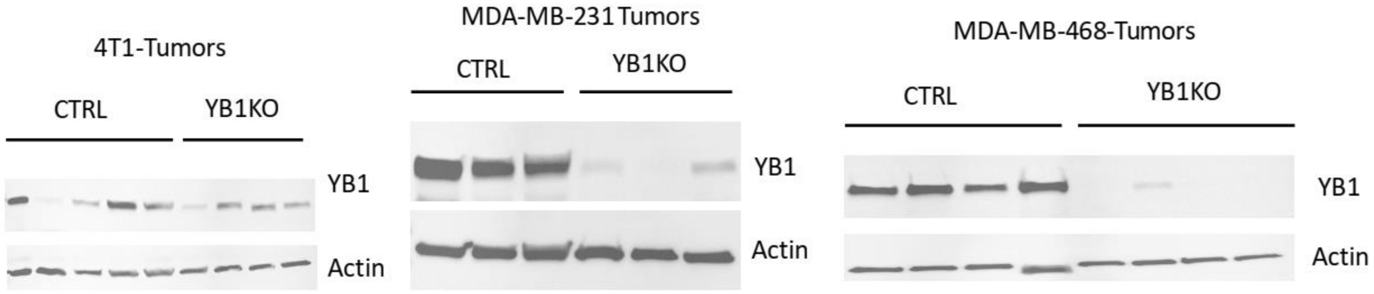

Figure S4. Western Blot analysis of protein lysates from tumors derived from the indicated parental (Ctrl) and their YB1-deficient (YB1KO) derivatives TNBC cell lines, that were probed with YB1 antibodies. β-Actin was used a loading control.

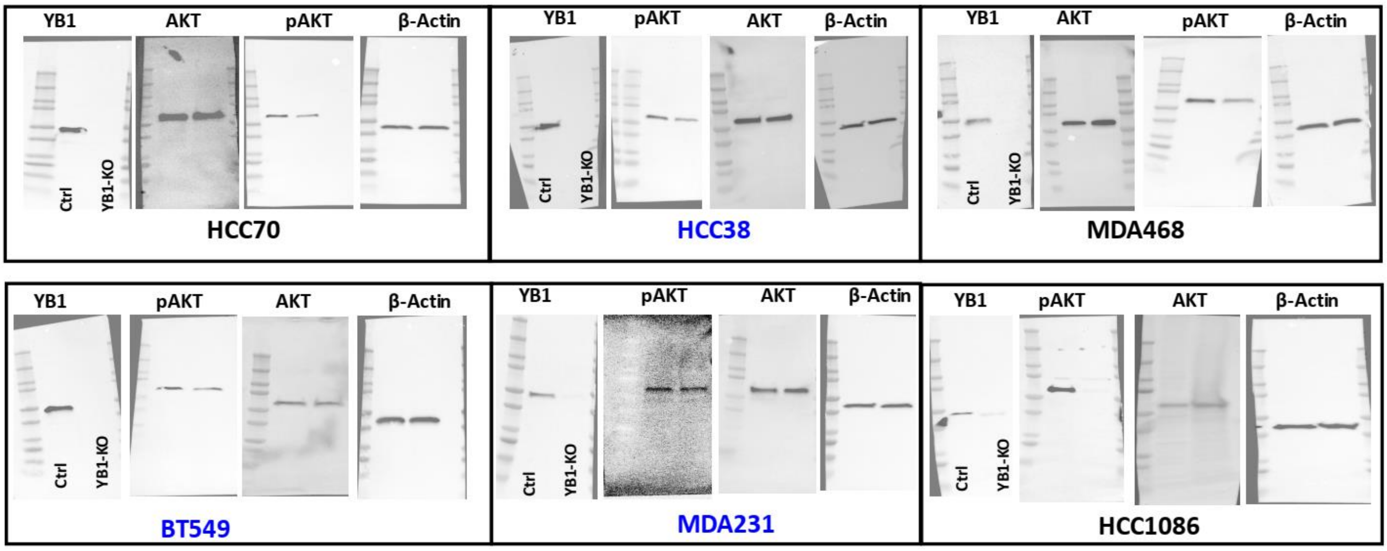

Figure S5. Uncropped Western blots for panel A of Figure 3.

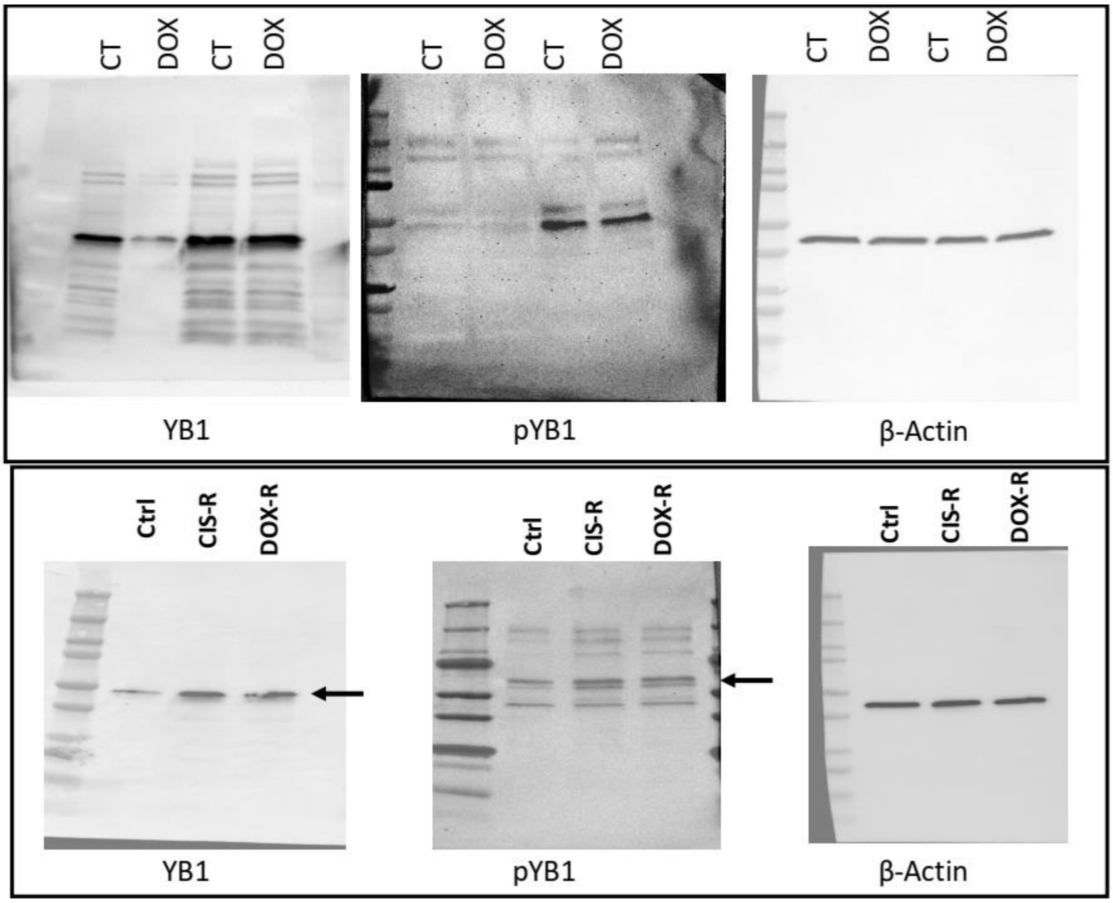

Figure S6. Uncropped WBs for panel E and panel F of Figure 6.
